# Current standards and novel concepts in thrombotic thrombocytopenic purpura

**DOI:** 10.3389/fmed.2026.1806030

**Published:** 2026-09-15

**Authors:** Johannes Bloehdorn, Mark S. Cragg, Martin Bommer

**Affiliations:** 1 Department of Hematology, Oncology, Infectious Disease and Palliative Care Medicine, Alb-Fils Klinikum GmbH, Göppingen, Germany; 2 School of Cancer Sciences, Faculty of Medicine, Centre for Cancer Immunology, University of Southampton, Southampton, United Kingdom

**Keywords:** ADAMTS13, caplacizumab, MAHA, TMA, TTP

## Abstract

Thrombotic thrombocytopenic purpura (TTP) is a rare and rapidly life-threatening thrombotic microangiopathy. Disseminated microvascular thrombosis and resulting ischemia are the critical events determining outcome, if left untreated. The pathogenic hallmark is a severe deficiency of the plasma metalloprotease ADAMTS13, resulting in impaired cleavage of ultra-large von Willebrand factor (vWF) multimers leading to platelet aggregation. The vast majority of TTP cases are acquired and immune-mediated, depleting ADAMTS13 through autoantibodies. In contrast, congenital deficiency of ADAMTS13 affects a minority of patients. Measurement of ADAMTS13 activity is the cornerstone for diagnosis and implementation of treatment. However, clinical prediction scores support early therapeutic decision-making when results are pending. Plasma exchange, immunosuppression and more recently, the anti-vWF nanobody caplacizumab, are the central pillars for treatment of immune-mediated TTP. Caplacizumab prevents microvascular thrombosis during the acute episode but does not address the autoimmune-mediated ADAMTS13 depletion, underscoring the importance of plasma exchange and immunosuppression. Congenital TTP was previously treated using ADAMTS13 replacement through regular plasma infusions, but provision of recombinant ADAMTS13 has recently transformed treatment in these patients. Despite major advances, relapse remains a persistent threat for patients, making structured, long-term follow-up necessary. Diagnostic advances may help improve relapse prediction and corresponding therapeutic management. This review summarizes current concepts in the pathophysiology, diagnosis, clinical management of TTP and highlights recent therapeutic advancements and future challenges.

## Introduction

Thrombotic thrombocytopenic purpura (TTP) is a rare and life-threatening thrombotic microangiopathy (TMA) with a unique pathophysiologic mechanism. It presents with sudden onset of systemic microvascular thrombosis, with subsequent increased consumption of platelets and microangiopathic hemolytic anemia (MAHA), mostly in previously healthy people. Depending on the organs involved, clinical presentation may vary broadly and commonly show non-specific symptoms.

Given the heterogeneous clinical picture, delaying or even missing the correct diagnosis is a major issue. Without treatment, the disease is typically fatal within a few days, and lethality rises sharply when diagnosis and initiation of therapy are delayed. In contrast, immediate diagnosis and treatment increase 3-month survival rates to above 90% and reduce long-term sequelae (1).

Thrombocytopenia with MAHA along with the hallmark findings of schistocytes and a negative direct antiglobulin (Coombs) test is present in the vast majority of cases and should prompt immediate action, including an appropriate diagnostic work-up and initiation of early treatment.

Thrombotic thrombocytopenic purpura was first described in 1924 by Eli Moschcowitz and further clinically defined by Amorosi and Ultman in 1966. It comprises two distinct forms; the acquired immune-mediated form (iTTP), known as Moschcowitz disease, and a hereditary (congenital) form (cTTP), known as Upshaw-Schulman syndrome. Although the disease pathomechanism is the same, their origins clearly differ (2).

In both forms, the disease results from a decrease in the plasma protease ADAMTS13 (a disintegrin and metalloproteinase with a thrombospondin type 1 motif, member 13). ADAMTS13 is responsible for cleaving ultra-large von Willebrand factor multimers (UL-vWF) and its critical decrease is necessary to initiate the TMA cascade.

While the vast majority of patients exhibit the defining features of iTTP and cTTP, some cases cannot be definitively classified into either category and may therefore be categorized as TTP with unknown pathophysiology (uTTP) (3).

Before effective therapy for TTP became available, an acute TTP episode was almost invariably fatal. Introduction of plasma exchange (PEX) revolutionized outcomes and reduced mortality.

Nowadays, with the addition of immunosuppression and caplacizumab along with PEX, mortality has been reduced to the low single-digit percentage range in experienced centers.

However, the success in treating acute TTP episodes has transformed TTP into a chronic disease, with relapses occurring in up to 40% of patients with iTTP (4), therefore requiring ongoing vigilance and follow-up.

Here, we provide a comprehensive overview of TTP diagnostic approaches and treatments with a perspective on therapeutic advancements and ongoing challenges.

## Epidemiology

Thrombotic thrombocytopenic purpura belongs to the so-called ultra-rare diseases and depending on the study and region, reported incidences range between 2 and 11 cases per million people annually (5).

Acquired TTP may affect all ages, but peaks around the fourth to sixth decade. The adult to child ratio is around 30:1 and there is a considerable female predominance with a female-to-male ratio roughly 2–3:1 (6, 7).

A study reporting results from the Oklahoma TTP-HUS registry analyzed incidences of TTP over a 6-year period involving affected children (<18 years old) and adults with severe acquired ADAMTS13 deficiency (<10% activity). The overall annual incidence rate of TTP was 2.17 per million, while the incidence in children was 0.09 per million and 2.88 per million in adults (7).

Notably, besides the considerably higher incidence in adults compared to children, the Oklahoma registry study also reported a higher incidence in patients of African descent (8.78 per million) compared with the white population (1.24 per million) (7).

These findings strongly suggest that genetic and environmental factors contribute to disease pathogenesis and should raise awareness in the respective clinical setting.

Incidences in Germany are reported as 1.47 per million (8) and in Canada as 1.8 per million (6) matching those of white US inhabitants.

Women also present at a younger age compared to men, peaking between 30–39 years (7) and 40–49 years (6) for women, and 40–49 years (7) and 60–69 years (6) for men, depending on the study.

The congenital form of TTP has been reported to be much less common than iTTP and current estimated prevalence is reported at 0.5–2.0 per million, based on the frequency of diagnosed patients (9).

However, the estimated genetic prevalence and carrier frequencies derived from genomic data and repositories have shown a considerable range, implying the possibility of underreported clinical presentation, regional differences or clinically asymptomatic cases (10–12).

A recent study conducting a comprehensive genetic epidemiology investigation using a large pool of genome and exome data from 807,162 individuals investigating the frequency of 140 documented pathogenic variants estimated the worldwide prevalence of cTTP to be 23 per million (13).

Due to the mode of inheritance, being autosomal recessive (only biallelic mutations show clinical manifestation), the likelihood increases in populations with multiple asymptomatic heterozygous carriers, such as in very small (stable) populations or those with consanguinity.

The degree of penetrance may also vary depending on the type of mutation and therefore homozygous patients may not necessarily become clinically apparent (5, 14).

Thrombotic thrombocytopenic purpura in neonates is in most cases congenital and cTTP mostly manifests through early childhood but shows variability according to the severity of ADAMTS13 absence or dysfunction.

About 38%–50% of cTTP manifests in early childhood mostly between 3.5 and 5 years of age (15, 16).

In some cases, first presentations may occur in adulthood, possibly triggered through accompanying stress such as pregnancy, trauma, surgery or infection.

Women with cTTP may be diagnosed more frequently as they may become clinically manifest with severe complications in the context of pregnancy (17).

Overall, these observations emphasize that TTP, while rare, shows considerable epidemiological and genetic heterogeneity, is likely underdiagnosed and shows marked clinical variability in presentation.

## Pathophysiology of TTP

As described above, the central pathogenic hallmark of TTP is a severe deficiency of ADAMTS13 metalloprotease activity and therefore reduced cleavage of UL-vWF multimers. In response to shear stress, inflammation or direct injury, UL-vWF is released from endothelial cells (Weibel-Palade bodies) and, to a much lesser extent, from platelets (α-granules) (18).

ADAMTS13, which is produced in liver stellate cells (19), rapidly cleaves UL-vWF at the Tyr1605-Met1606 bond in the A2 domain of vWF and prevents excessive platelet aggregation. The vWF adopts an increasingly exposed conformation, amenable for cleavage, when shear stress increases (20, 21).

In TTP, the activity of ADAMTS13 is greatly reduced (<10% of normal activity) and insufficient to maintain adequate cleavage to prevent formation of highly adhesive UL-vWF. As a consequence, these UL-vWF bind platelets, especially in the microcirculation where high shear stress promotes their release, resulting in widespread microvascular platelet-rich thrombosis (22).

In contrast to disseminated intravascular coagulation (DIC) where the initial pathophysiologic mechanism promotes formation of fibrin-rich clots with consumption of fibrinogen and coagulation factors, TTP is characterized by platelet-rich thrombi, largely stable levels of fibrinogen and coagulation parameters (22).

Thrombotic thrombocytopenic purpura is increasingly viewed within a multi-hit disease model.

Although severe ADAMTS13 deficiency is central to disease pathogenesis, profound deficiency alone may not always be sufficient to cause symptomatic disease (23, 24).

Instead, clinical manifestation of TTP is thought to arise from a convergence of severe ADAMTS13 depletion, most commonly due to loss of immune tolerance with anti-ADAMTS13 autoantibody formation, and additional precipitating “second hits,” involving endothelial activation and UL-vWF release.

Such triggers may include infection, vaccination, inflammation, pregnancy and surgery (25–29).

Several factors increase the susceptibility to ADAMTS13 autoimmunity, including specific HLA class II alleles (30).

Importantly, some patients show prolonged periods of critically reduced ADAMTS13 activity before clinical relapse (23, 24). This supports the concept that “second hits” are required for the transition from biochemical relapse to overt clinical disease with symptomatic microvascular thrombosis.

This framework has practical implications and strengthens the rationale for structured remission monitoring and individualized relapse-prevention strategies, particularly in high-risk settings such as pregnancy planning, intercurrent infection and preemptive immunosuppression when ADAMTS13 declines.

### Immune-mediated ADAMTS13 deficiency (iTTP)

Immune-mediated ADAMTS13 deficiency comprises the vast majority of presentations at roughly 95% of cases in adults.

Patients develop autoantibodies against ADAMTS13, mostly IgG and specifically IgG4, which neutralize the vWF-cleaving activity and promote ADAMTS13 clearance from the plasma (31–34).

Approximately 10%–20% of patients exhibit IgA- or IgM autoantibodies. Higher anti-ADAMTS13 antibody titers of the IgA type have been correlated with a more severe acute presentation, while IgG4 presence has been associated with an increased risk of relapse (31, 35, 36).

The initiating event for these autoantibodies is largely unknown but molecular mimicry in the context of infections and genetic predispositions (HLA class II alleles) may contribute. While no specific infectious trigger has been identified, associations have been reported for influenza, *Helicobacter pylori*, *Legionella pneumophila*, hepatitis C, human immunodeficiency virus, cytomegalovirus and SARS-CoV-2 (25, 27, 37–42).

Recent findings also highlight that, in addition to direct enzymatic inhibition, enhanced clearance of ADAMTS13 represents a major pathogenic mechanism in iTTP. Autoantibody binding can lead to the formation of immune complexes that are rapidly removed from the circulation via Fc receptor-mediated pathways, resulting in profound ADAMTS13 deficiency (43). This mechanism may explain the severe reduction in ADAMTS13 antigen levels observed in many patients and underscores the importance of considering both functional inhibition and antigen clearance in disease pathophysiology.

Further, the pathogenic immune response in iTTP is characterized by polyclonal autoantibodies against all domains of ADAMTS13, however, in the vast majority of cases antibodies against the cysteine-rich and spacer domains are found (44–47).

Moreover, conformational change in ADAMTS13 has been identified as a key feature of iTTP. ADAMTS13 circulates in an inactive closed conformation where C-terminal CUB domains interact with the spacer domain (48). After binding to specific sites on vWF, ADAMTS13 adopts an open conformation and becomes enzymatically active (49).

This open conformation is evident during acute iTTP and appears also to be mediated by autoantibody binding (50). In contrast, the closed conformation is found in iTTP patients in remission, but reversion to the open conformation may signal an imminent relapse (51). Assays to detect ADAMTS13 conformational state as a predictive tool for relapse likelihood are under investigation.

Beyond the classical paradigm of immune-mediated ADAMTS13 deficiency, emerging data suggest that additional mechanisms may contribute to the clinical phenotype of iTTP. In particular, patients with acute iTTP may show a low absolute immature platelet count (A-IPC) at presentation, a characteristic that may also help to distinguish iTTP from other TMAs (52–54).

These findings are in contrast to the compensatory increase in thrombopoiesis typically observed in consumptive thrombocytopenias and suggest an insufficient compensatory thrombopoietic response in iTTP, with possible impairment of immature platelet production. Notably, recent data suggest that low A-IPC may potentially be indicative of the severity of presentation, since low A-IPC was found in iTTP patients with high ADAMTS13 inhibitor levels (55).

Given these pillars within the pathogenic sequence for initiation and relapse of iTTP, the immune axis represents a major focus of therapeutic intervention in iTTP and has conceptually been targeted with immunosuppression, which targets different points within the amplification of immune responses and the generation of autoantibodies.

### Congenital ADAMTS13 deficiency (cTTP)

Congenital ADAMTS13 deficiency comprises a smaller cohort of patients with inborn dysfunction or absence of ADAMTS13 due to gene mutations, through autosomal recessive inheritance.

Several hundred mutations have been described, including missense, nonsense, and splice-site variants, among others (13), and can manifest with varying degrees of severity depending on their functional effects on the translated protein.

For symptomatic disease, homozygous or compound heterozygous mutations are necessary. Patients with heterozygous mutations are clinically not affected but may have a higher risk of ischemic events (9, 13, 15, 56–63).

Regional differences for the distribution of genotypes are described. For example, patients of European ancestry frequently carry the *ADAMTS13* mutations p.R1060W (patients with adult-onset cTTP, particularly pregnancy associated first presentation) and c.4143_4144dupA (found in clusters in Scandinavia, around the Baltic Sea and central Europe) (11, 15, 28, 63–67).

Notably, a recent study provided a comprehensive overview of the distribution of the known pathogenic variants with regard to ethnicity. It also suggested a much higher number of variants (618 novel variants with pathogenic features) beyond what had been previously reported. However, the pathogenic significance remains incompletely characterized and requires further investigation (13).

The median age at presentation in cTTP is 4.5 years, with a wide range from birth to 69.8 years, reflecting the marked clinical heterogeneity of the disease (63).

Consistent with this variability, early-onset and adult-onset forms can be distinguished (57). Patients with early-onset disease may become clinically apparent at birth, presenting with severe hemolysis and neonatal jaundice that often necessitate whole-blood exchange transfusion; however, the diagnosis of cTTP is frequently delayed for several years, as reported in independent cohorts from Japan and Norway (11, 57).

Patients are frequently diagnosed during early childhood after events triggering first cTTP manifestation, such as infection, trauma or surgery. In contrast, the adult-onset form remains clinically silent until additional triggers, most commonly pregnancy, lead to manifestation (28, 29, 67, 68).

Patients with cTTP may show single or recurrent TMA episodes with exacerbation depending on the initiating trigger. Besides the type of mutation, as mentioned above (28), residual protein function has been correlated with time to onset and relapse risk. ADAMTS13 activity of below 3% strongly correlates with early disease manifestation (residual activity <2.74%) and more frequent episodes requiring therapeutic intervention (residual activity <1.61%) (62).

Irrespective of these factors, single genetic variants can present with a broad spectrum of clinical manifestations, suggesting that other factors significantly influence underlying pathogenicity.

Treatment in patients with cTTP therefore differs from that in patients with iTTP with regard to the underlying cause and predominantly aims to replenish the missing or dysfunctional ADAMTS13 (69).

### TTP with unknown pathophysiology (uTTP)

Despite the majority of patients presenting with the well-established hallmarks of iTTP and cTTP, a proportion of patients are not categorized as clearly (3).

Such patients may present with low ADAMTS13 activity but the absence of an identifiable inhibitor or gene mutation (70, 71).

Such findings have predominantly been described in association with conditions like hepatic disorders, infections, malignancies and organ transplantations, and may comprise defective secretion, proteolytic degradation or catalytic inhibition (72, 73).

A unifying feature in these cases is the detection of ADAMTS13 in the closed conformation, suggesting a distinct pathophysiology. As the mechanism remains unidentified, the combination of characteristics has therefore been summarized as uTTP (70).

A recent retrospective study highlighted the possibility of initial misdiagnosis of iTTP due to undetected antibodies at initial presentation but detectable levels during the follow-up period.

However, this study identified a considerable portion of patients never showing detectable inhibitor levels and presenting with distinct clinical characteristics (74).

## Clinical presentation and diagnostic work-up

### Clinical presentation

Thrombotic thrombocytopenic purpura usually manifests in a highly dynamic fashion over a few days or weeks. Patients mostly present with non-specific symptoms related to microangiopathic hemolysis and diffuse fluctuating ischemia in various organs resulting in fatigue, muscular weakness and dizziness (58, 75). On physical examination, key findings include pallor, jaundice, tachycardia, petechiae or bruising and possibly mild splenomegaly. Irrespective of the underlying cause for diminished ADAMTS13 activity, the degree of microvascular thrombosis determines the severity of clinical manifestation, most prominently affecting the brain, heart, and kidneys.

Analysis of the clinical manifestations and leading symptoms in patients with iTTP included in the Oklahoma registry studies (76, 77) identified gastrointestinal symptoms amongst the most frequent with presence in 69% of patients (76).

Neurological symptoms may be even more common but can range from mild (headache, confusion) to severe manifestations (coma, stroke, seizures) and often fluctuate (78).

Any neurological change in a patient with thrombocytopenia and MAHA should raise immediate suspicion of TTP (77, 79).

In acute TTP, neurologic symptoms may occur despite normal brain MRI imaging and on the other hand, imaging abnormalities do not reliably parallel clinical severity. Imaging findings are heterogeneous, with posterior reversible encephalopathy syndrome (PRES) reported particularly in severe cases, however, extensive imaging abnormalities may be reversible and not necessarily associated with poor neurologic outcome. Frontal white matter lesions have been associated with later cognitive impairment; anxiety and depression are also common, including patients without neurologic involvement at presentation (80).

Renal involvement is also common but typically mild compared to HUS, with slightly elevated creatinine, hematuria, and proteinuria. Severe renal failure requiring dialysis is uncommon and should prompt consideration of other diagnoses. Serum creatinine levels were less than 1.5 mg/dL in 47.5% of patients in the Oklahoma registry analysis (77, 81).

Signs of bleeding and muscle weakness are common, whereas fever is less frequent; high fever should prompt evaluation of alternative diagnoses such as sepsis (76, 77).

Lymphadenopathy or significant organomegaly is generally absent and should prompt extended diagnostics.

Cardiac involvement is clinically relevant and myocardial ischemia is a contributor to acute mortality; therefore, cardiac monitoring (laboratory and ECG) is indicated in the acute phase. Further, signs of heart failure may be present and accompanied by persistent arrhythmia if cardiac involvement is severe (76, 82, 83).

As discussed above, the five classic characteristics of TTP, the so-called TTP pentad, described in 1966 by Amorosi and Ultman, consisting of hemolytic anemia, thrombocytopenia, neurologic symptoms, renal dysfunction and fever are rarely (<5%) all present at initial patient contact (76, 77).

Symptoms in cTTP are highly similar to iTTP but may differ in frequency. Stroke or transient ischemic attacks are frequent in cTTP patients, while myocardial infarction is less common (63). Severe renal impairment may occur (57, 63).

### Diagnostic and laboratory work-up

Hallmark laboratory findings (Table 1) in TTP are MAHA and thrombocytopenia. Schistocytes are often seen with >1% of red blood cells, but even low numbers in the appropriate context should prompt evaluation for TMA.

**TABLE 1:** Diagnostic rationale and key laboratory testing in patients with suspicion of thrombotic thrombocytopenic purpura (TTP) (adapted from (84)).

Diagnostic rationale	Key laboratory testing	Diagnostic implication
TMA phenotype	CBC; blood smear (schistocyte percentage); LDH; haptoglobin; reticulocytes	Confirms MAHA; a leukoerythroblastic smear suggests marrow infiltration
Thrombocytopenia	Platelet count	MAHA with thrombocytopenia strongly supports diagnosis of TMA
Renal involvement	Serum creatinine; urine testing for proteinuria	Severe renal failure favors HUS over TTP
Hemolysis pattern/hepatopathy	LDH; haptoglobin; bilirubin (direct/indirect); AST/ALT; ALP/GGT	Supports intravascular hemolysis; liver enzymes aid interpretation and help exclude hepatopathy
Exclude autoimmune hemolysis	DAT (direct antiglobulin test)	DAT usually negative in TMA; DAT positivity does not exclude TMA; Evans syndrome typically lacks schistocytes
Differentiate from DIC / hyperfibrinolysis	PT/INR; aPTT; fibrinogen; AT; D-dimer; vWF:Ag and activity	Distinguishes primary TMA from DIC or cancer-associated hyperfibrinolysis
Confirm TTP	ADAMTS13 activity and inhibitor testing (sample or store before plasma-containing therapy)	ADAMTS13 activity <10% and presence of an inhibitor confirms iTTP
Infection-associated HUS/TMA	Shiga toxin in stool; blood cultures; HIV/EBV/CMV PCR	Identifies STEC-HUS; *S. pneumoniae*-associated HUS; virus-associated TMA
Complement-mediated TMA	sC5b-9; C3; C4; anti-FH Ab	Supports complement-mediated TMA (cmTMA/aHUS)
Autoimmune-associated TMA	ANA; aPL	Suggests autoimmune disease-associated TMA
Biopsy for validation/histology	Bone marrow biopsy (cytology and IHC); kidney/skin biopsy (C5b-9 IHC)	Identifies bone marrow carcinomatosis; complement-mediated nephropathy/TMA
Hereditary TMA	Genetic testing (*ADAMTS13*; *CFH*; *CFI*; *MCP*; *C3*; *DGKE*)	Confirms congenital TTP or cmTMA

aHUS, atypical hemolytic uremic syndrome; ALP, alkaline phosphatase; ANA, antinuclear antibodies; aPL, antiphospholipid antibodies; aPTT, activated partial thromboplastin time; AT, antithrombin; CBC, complete blood count; CFH, complement factor H; CFI, complement factor I; cmTMA, complement-mediated thrombotic microangiopathy; DAT, direct antiglobulin test; DGKE, diacylglycerol kinase epsilon; DIC, disseminated intravascular coagulation; EBV, Epstein–Barr virus; FH Ab, factor H antibodies; GGT, γ-glutamyl transferase; HUS, hemolytic uremic syndrome; IHC, immunohistochemistry; LDH, lactate dehydrogenase; MAHA, microangiopathic hemolytic anemia; MCP, membrane cofactor protein; PCR, polymerase chain reaction; PT/INR, prothrombin time/international normalized ratio; sC5b-9, soluble terminal complement complex; STEC, Shiga toxin–producing *Escherichia coli*; TMA, thrombotic microangiopathy; TTP, thrombotic thrombocytopenic purpura.

Hemoglobin is often reduced (often around 7–10 g/dL or lower) and platelet counts often decrease to <30 × 10^9^/L. Lactate dehydrogenase (LDH) is markedly increased, indirect bilirubin elevated, haptoglobin decreased or undetectable and reticulocytes are increased. The direct antiglobulin (Coombs) test is usually negative, but a positive test does not rule out TTP. Hemoglobinuria may be present if hemolysis is excessive (84–86).

Initiation of thrombosis is mediated through excessive aggregation of thrombocytes without considerable activation of the coagulation cascade. Fibrinogen levels are therefore typically normal, along with prothrombin time (PT) and aPTT, while D-dimer levels may be slightly increased. These findings help to distinguish TTP from DIC or other coagulopathies, which are characterized by low fibrinogen, markedly increased D-dimers and prolonged PT (84, 85).

Creatinine is normal or mildly elevated. Troponin may be elevated, indicating myocardial ischemia.

ADAMTS13 activity <10% confirms the diagnosis and most patients show <5% activity. Testing for an ADAMTS13 inhibitor is essential and confirms iTTP. In cTTP, genetic testing for ADAMTS13 mutations is the method of choice (9, 85, 86).

It is of utmost importance that blood samples for ADAMTS13 testing are drawn before plasma-containing treatment is initiated, as ADAMTS13 within the infused plasma will confound test results. Therefore, once TTP is suspected, citrated plasma samples for ADAMTS13 assays should be collected and ideally frozen. Modern ADAMTS13 assays can provide results within 1–2 days in many centers, some specialized laboratories have rapid assays with turnaround of <8 h (84, 85).

Pregnancy testing is recommended in premenopausal women, as pregnancy can trigger TTP. Screening for underlying diseases that may mimic or trigger TTP includes lupus and HIV. Basic screening for underlying cancer is recommended as TMAs can occur in a paraneoplastic fashion. While rare, some drugs may also induce TMA and can be considered in the specific context. The contextualized approach to laboratory testing and differential diagnosis is summarized in Tables 1 and 3 (84, 85).

**TABLE 2:** Parameters for calculating the PLASMIC score (88) and FRENCH score (87) (adapted from (84)).

Laboratory or clinical variable	PLASMIC score (1 point each)	FRENCH score (modified) (1 point each)
Thrombocytopenia	Platelets <30 × 10^9^/L	Platelets <30 × 10^9^/L
Hemolysis	Reticulocytes >2.5% or indirect bilirubin >2 mg/dL or undetectable haptoglobin	Not included
Mean corpuscular volume (MCV)	MCV <90 fL	Not included
Kidney function	Creatinine <2.0 mg/dL	Creatinine ≤ 2.26 mg/dL
Coagulation status	INR <1.5	Not included
Cancer history	No active malignancy	Score excludes patients with malignancy
Transplant history	No history of solid organ or stem cell transplant	Score excludes transplant patients
Autoantibodies	Not included	In original FRENCH score: ANA positive
Score range	0–7 points	0–2 points
Likelihood of severe ADAMTS13 deficiency (iTTP)	0–4: low (0%–4%) 5: intermediate (5%–24%) 6–7: high (62%–82%)	0: low (2%) 1: intermediate (70%) 2: high (94%)

The PLASMIC score comprises seven variables (**P**latelet count, hemo**L**ysis variable, absence of **A**ctive cancer, absence of **S**tem cell/**S**olid organ transplant, **M**CV, **I**NR, **C**reatinine) while the FRENCH score utilizes just two variables. ANA, antinuclear antibodies; INR, international normalized ratio; LDH, lactate dehydrogenase; MCV, mean corpuscular volume.

**TABLE 3:** Differential diagnoses of thrombotic microangiopathy (TMA)-like diseases considered in the clinical and diagnostic work-up (adapted from (84)).

Condition	Pathophysiology	Key features differentiating from TMA	Treatment
Infection-associated				
Endocarditis Sepsis with DIC Malaria, babesiosis Hemorrhagic fever/viral infections	Bacteremia, sepsis, valvular vegetations Consumptive coagulopathy Parasite-induced hemolysis Dengue virus, filoviruses, Puumala virus, SARS-CoV-2	Blood cultures, TEE Blood cultures, ↑ procalcitonin, ↓ fibrinogen, ↑ D-dimers Parasites on peripheral blood smear microscopy Mostly lacks classic MAHA, exposure/travel history	Targeted antibiotic therapy Sepsis management, antibiotics Antiparasitic therapy Supportive therapy
Pregnancy-associated				
Acute fatty liver of pregnancy (AFLP) (Pre-)eclampsia/HELLP syndrome	Hereditary defects of lipid metabolism Endothelial dysfunction	Hypoglycemia, ↑ ALT/AST, ↑ bilirubin, ↓ AT, ↓ clotting factors Hypertension, ↑ sFlt-1/PlGF ratio	Prompt delivery, supportive therapy Delivery, supportive therapy
Malignancy-associated				
Disseminated or aggressive malignancy Trousseau syndrome/hypercoagulability	Hyperfibrinolysis, DIC, marrow infiltration Paraneoplastic thrombosis, endothelial damage	↓ Fibrinogen, blasts/leukoerythroblastic picture on blood smear ↑↑ D-dimers, sometimes ↑ CK (ischemia)	Treat malignancy and coagulopathy Treat malignancy, start anticoagulation
Immune-mediated				
Evans syndrome (idiopathic or secondary) Hemophagocytic lymphohistiocytosis (HLH) Catastrophic antiphospholipid syndrome Scleroderma renal crisis Heparin-induced thrombocytopenia (HIT) ABO-incompatible transfusion	ITP and Coombs-positive AIHA, e.g. secondary to CLL/HIV/SLE Macrophage-mediated hemophagocytosis Endothelial damage, diffuse thrombosis Intimal proliferation and luminal obliteration Anti-PF4 antibody-mediated platelet activation Massive intravascular hemolysis with complement activation	Direct Coombs test positive, no schistocytes ↑↑ Ferritin, ↑ triglycerides, hemophagocytosis (microscopy) Anti-cardiolipin/anti-β2GPI antibodies, lupus anticoagulant, ↑ aPTT Severe hypertension, anti-RNA polymerase III antibodies Heparin exposure, 4Ts score, HIT immunoassay Direct Coombs test positive, received transfusion	Immunosuppression/specific therapy HLH-directed therapy (HLH protocols) Anticoagulation, immunosuppression Aggressive BP control, ACE inhibitor Prompt non-heparin anticoagulation Supportive, hemolysis/DIC management
Metabolic disorder				
Severe vitamin B_12_ deficiency (“pseudo-TTP”) Wilson disease	Ineffective erythropoiesis, hemolysis-like/neurologic features ATP7B mutation, hemolysis due to copper release	↓ Reticulocytes, ↑↑ LDH, ↑ methylmalonate, macrocytic anemia ↓ Ceruloplasmin, ↑ urinary copper	Vitamin B_12_ replacement Copper chelation
Inborn/acquired defects				
Hemoglobinopathies (e.g. sickle cell crisis) Paroxysmal nocturnal hemoglobinuria (PNH)	Structural or functional hemoglobin/erythrocyte defects Acquired deficiency of GPI-anchored complement inhibitors	RBC morphology, Hb electrophoresis, genetics Flow cytometry showing GPI-anchor deficiency	Disease-specific therapy Complement inhibition
Other				
Generalized seizures/(malignant) hyperthermia Malignant hypertension Heart valve-induced hemolysis	Rhabdomyolysis Severe hypertension causing microvascular endothelial injury Mechanical erythrocyte fragmentation/platelet consumption	History of seizures/hyperthermia, ↑↑ CK Severe hypertension with end-organ injury Valve prosthesis or valve defect, TEE	Supportive care/aggressive hydration Aggressive BP control Correction of valve defect

ACE, angiotensin-converting enzyme; ADAMTS13, a disintegrin and metalloproteinase with thrombospondin type 1 motif member 13; AFLP, acute fatty liver of pregnancy; AIHA, autoimmune hemolytic anemia; ALT, alanine aminotransferase; anti-PF4, anti-platelet factor 4; AST, aspartate aminotransferase; AT, antithrombin; BP, blood pressure; CK, creatine kinase; CLL, chronic lymphocytic leukemia; DIC, disseminated intravascular coagulation; GPI, glycosylphosphatidylinositol; Hb, hemoglobin; HELLP, hemolysis, elevated liver enzymes, and low platelets; HIT, heparin-induced thrombocytopenia; HIV, human immunodeficiency virus; HLH, hemophagocytic lymphohistiocytosis; ITP, immune thrombocytopenia; LDH, lactate dehydrogenase; MAHA, microangiopathic hemolytic anemia; PNH, paroxysmal nocturnal hemoglobinuria; aPTT, activated partial thromboplastin time; RBC, red blood cell; SLE, systemic lupus erythematosus; sFlt-1/PlGF, soluble fms-like tyrosine kinase-1/placental growth factor; TEE, transesophageal echocardiography; TMA, thrombotic microangiopathy.

### Diagnostic approach and TTP risk scores

Because delayed treatment is associated with high mortality, clinicians must often decide to treat presumptively for TTP before ADAMTS13 results are available.

To better estimate the likelihood of TTP and provide a standardized basis for deciding on treatment initiation, clinical scoring systems have been explored, of which two were validated more broadly.

The French TMA Reference Center Score (FRENCH score) was developed in 2010 (87) and initially identified three parameters for the prediction of TTP likelihood, but was adapted later to incorporate just two variables.

In 2017 a larger cohort was analyzed, comprising patients with TMA and a possible diagnosis of TTP, all presenting to one of the centers from the Harvard TMA Research Collaborative. Here the authors identified seven variables to accurately identify TTP patients and established the so-called PLASMIC score (88).

Both scoring systems assign one point for each of the identified variables and a corresponding risk category for the probability of TTP with severe ADAMTS13 deficiency (Table 2). A high score provides a strong basis to assume TTP. However, attention must still be paid as TTP cases may have lower scores and if clinical suspicion is high, treatment should be initiated irrespective of the score results.

Notably, due to the unusual presentation with moderate or severe kidney injury, presence of mild impairment or even normal function raises the suspicion of TTP and increases the calculated likelihood when using the PLASMIC or FRENCH scoring systems (Table 2).

In 2023, Park et al. assessed the accuracy of three risk scores, including the Bentley score (89) developed in 2010, in a cohort of 145 patients suspected of TMA. The PLASMIC score showed higher negative and positive predictive values compared to the FRENCH and the Bentley score, while the Bentley score was the least accurate.

The authors also suggested a modified PLASMIC score (cut-off >6) by adding the ratio of LDH/upper limit of normal (LDH/ULN). The modified PLASMIC score increased the specificity, PPV, and accuracy to 97.0%, 92.3%, and 92.4%, respectively (90).

The PLASMIC score is the most extensively validated scoring tool, shows superior sensitivity and specificity, and broader consideration of individual aspects in the clinic (85, 91).

However, the FRENCH score holds practical value in some situations, especially when a fast evaluation based on very few values is needed, for example in emergency settings or before other variables are available.

In cases with lower likelihood but sufficient clinical suspicion, e.g., if there is an intermediate probability of TTP, such as represented by PLASMIC score of 5 or FRENCH score of 1, initiation of PEX and immediate accompanying therapy is the safest route in clinical management (Figure 1) with outcomes being markedly better with early treatment (85, 92).

**FIGURE 1:**
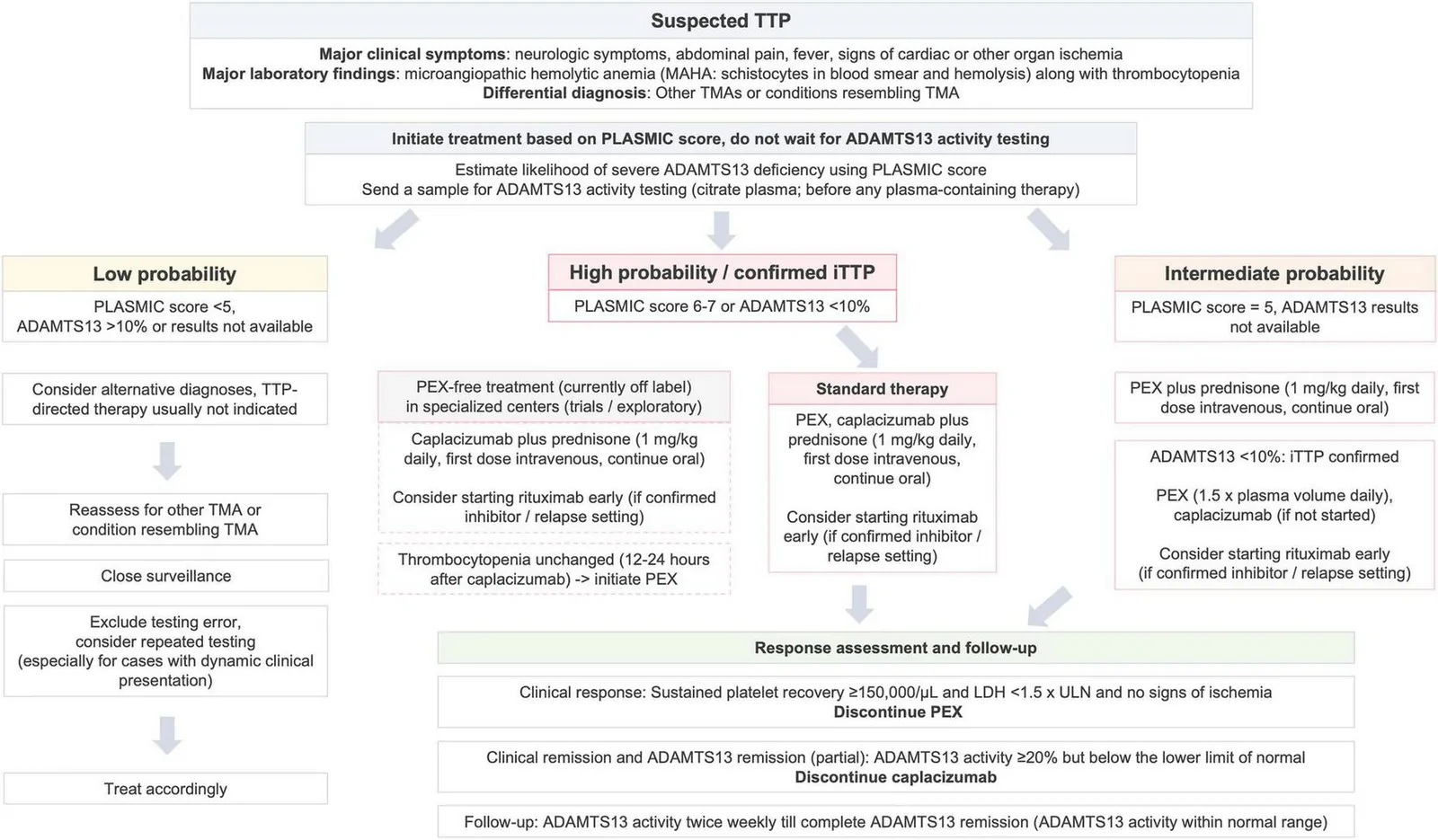
Diagnostic and therapeutic algorithm incorporating the PLASMIC score for initial stratification. ADAMTS13, a disintegrin and metalloproteinase with thrombospondin type 1 motif member 13; iTTP, immune thrombotic thrombocytopenic purpura; LDH, lactate dehydrogenase; MAHA, microangiopathic hemolytic anemia; PEX, plasma exchange; TMA, thrombotic microangiopathy; TTP, thrombotic thrombocytopenic purpura; ULN, upper limit of normal.

In comparison, risk and consequences of treating a TMA with PEX and adjunctive therapy, which subsequently turns out to have normal ADAMTS13 activity, are comparably low in contrast to delayed treatment of a TTP patient.

In contrast, a patient with a PLASMIC score of 4 or less has a higher likelihood of an alternative diagnosis, such as DIC from sepsis or HUS from *Escherichia coli*, which is why the omission of PEX may be justified until ADAMTS13 testing is finalized (Tables 1–3) (85).

It is important to note that emerging biomarkers such as immature platelet fraction (IPF) and especially A-IPC have shown promise as adjunctive tools in the evaluation of suspected iTTP (52–54). These parameters may support diagnosis, help distinguish iTTP from other thrombotic microangiopathies and provide information on response to PEX.

They may be especially useful in situations where ADAMTS13 testing is not rapidly available or when clinical prediction tools like the PLASMIC score deliver indeterminate or potentially misleading results.

Especially A-IPC has been shown to be useful for differentiating the underlying pathology, discrepant PLASMIC scores and response to treatment (54, 93).

Since IPF and A-IPC can be easily obtained from modern hematology analyzers, they represent an attractive additional source of information. Nevertheless, these biomarkers remain complementary and are not yet validated sufficiently to replace ADAMTS13 testing in routine practice and prospective validation is still needed.

### Differential diagnosis of thrombotic microangiopathies

If ADAMTS13 testing is unavailable during initial diagnostic work-up or activity is >10%, other TMA causes should be considered. Key alternative causes of MAHA and thrombocytopenia and conditions resembling TMA are summarized in Tables 1, 3 (84).

In HUS, renal failure predominates over neurologic involvement, unlike in TTP. HUS is most often caused by Shiga-toxin-producing *E. coli* (STEC, usually O157:H7), called “typical” HUS. Typical presentation consists of bloody diarrhea in children followed by acute kidney injury, MAHA and thrombocytopenia. Although less common, adults can develop STEC-HUS, often with a more severe course. ADAMTS13 activity is usually ≥30% or normal. Stool culture and PCR for *E. coli*/Shiga toxin confirm the diagnosis. Initial treatment is supportive but may involve dialysis if needed. Antibiotics should be avoided because they may increase Shiga toxin release. Because ADAMTS13 is normal, PEX is not indicated (84).

Rarely, HUS follows pneumococcal infection (SP-HUS), typically in young children. It involves neuraminidase stripping of sialic acid from cell surface and subsequent T-antigen exposure on RBCs, platelets and endothelial cells, which is recognized by the immune system. Treatment includes antibiotics and supportive care (dialysis/transfusions as needed) (84).

Atypical HUS (complement-mediated TMA) is driven by uncontrolled complement activation due to genetic defects in complement regulators (e.g., factor H, I, CD46) or autoantibodies (e.g., anti-factor H). Atypical HUS may mimic TTP, but has severe renal involvement and fewer neurologic symptoms.

It is critical to distinguish aHUS from STEC-HUS, TTP and other secondary TMAs (e.g., malignancy- or drug-associated). Genetic or antibody confirmation [e.g., complement genes, DGKE (diacylglycerol kinase ϵ)] is not required for an aHUS diagnosis (94, 95).

Inherited complement defects are more common than acquired alterations. Factor H mutations are the most frequent among the known complement gene mutations. C5 inhibitors (eculizumab or ravulizumab) are the treatment of choice. ADAMTS13 activity in aHUS is >10% and specialized testing (complement levels, genetic analysis) is needed, but therapy should begin on clinical suspicion of aHUS (after excluding TTP with ADAMTS13) (94, 95).

Malignant hypertension can cause endothelial damage with fibrin deposition, leading to ischemia and MAHA. Here, aggressive blood pressure control is the treatment of choice.

In pregnancy, HELLP syndrome (*Hemolysis, Elevated Liver Enzymes, Low Platelets*) can mimic TTP/HUS and typically manifests in the last trimester or peripartum. Distinguishing HELLP from TTP/HUS can be difficult if MAHA is pronounced. Very high transaminases (AST/ALT >2–3 × upper limit of normal), may favor HELLP (sometimes with concurrent coagulopathy). TTP often occurs earlier in pregnancy. Schistocytes on blood smear strongly support a pregnancy-related TMA (96).

Delivery is the definitive treatment for HELLP. By contrast, suspected TTP in pregnancy requires immediate PEX. However, ADAMTS13 testing, while vital for distinguishing TTP from HELLP, is time-consuming, and results are often available after delivery (86, 97). As both conditions require prompt treatment, PEX and concurrent delivery may be conducted in certain situations (96, 97).

Registry data suggest TTP occurs more likely antepartum, whereas aHUS (cmTMA) is more common postpartum. In congenital TTP or aHUS, pregnancy can trigger the disease (97, 98).

Acute fatty liver of pregnancy (AFLP) causes acute liver failure with DIC, hemolysis and thrombocytopenia. It presents with severe hepatic dysfunction and hypoglycemia, unlike TTP (96).

Catastrophic antiphospholipid syndrome (CAPS) causes widespread thromboses, MAHA, consumptive thrombocytopenia and DIC-like labs, often in patients with known autoimmune disease or antiphospholipid antibodies. Treatment of CAPS involves managing the underlying condition and anticoagulation (especially after thrombosis) (99).

Also, certain drugs can cause TMA. Best examples comprise chemotherapies (e.g. mitomycin C, gemcitabine, vascular endothelial growth factor (VEGF) inhibitors) (100) and immunosuppressants (cyclosporine and tacrolimus), showing dose-dependent endothelial toxicity (101). Stopping the drug administration usually leads to recovery.

Transplant-associated TMA (taTMA) can occur after stem cell or organ transplant (often associated with graft-versus-host disease, calcineurin inhibitors and infections). The prognosis is poor and optimal treatment remains undefined, although complement inhibitors may help (102, 103).

Malignancy-associated TMA is rare. Drug-induced TMA should be considered in the differential diagnosis when the underlying malignancy is being actively treated with TMA-causing therapeutics. Extensive marrow involvement or circulating tumor cells can induce a TMA-like picture. The prognosis is very poor and chemotherapy may worsen the situation (104).

Many conditions can mimic or overlap with TTP and laboratory/clinical features alone may be misleading. Scoring systems (PLASMIC and FRENCH score) and the clinical context should guide treatment decision (85, 92).

If ADAMTS13 activity is <10%, treatment for TTP should be initiated or augmented with caplacizumab if plasma exchange has already been started. In cases with normal or moderately reduced ADAMTS13 activity, readjusting the diagnostic and therapeutic strategy is warranted (85, 92).

## Management of immune-mediated TTP (iTTP)

With TTP representing a medical emergency, treatment should be initiated immediately when TTP is suspected.

Primary treatment principles in iTTP target all pathogenic mechanisms at once through a combined therapeutic approach which addresses (1) the removal and suppression of the existing anti-ADAMTS13 autoantibodies and UL-vWF through PEX along with the autoantibody producing B cells by using steroids and rituximab; (2) increasing functional ADAMTS13 levels by using PEX; (3) preventing UL-vWF-mediated clotting of platelets using caplacizumab (summarized in Figure 1).

### Therapeutic plasma exchange

The mainstay of TTP treatment has historically been and currently remains PEX (105), which was first used as an effective treatment after findings of successful whole blood exchange (106–108). PEX and steroids were subsequently described as an effective treatment in a trial of 108 patients (109).

Plasma exchange is conducted by exchanging the plasma from the patient and thereby removing both the disease-causing anti-ADAMTS13 autoantibodies and UL-vWF. The concurrently replaced donor fresh frozen plasma (FFP) contains functional ADAMTS13 which cleaves the UL-vWF, thereby stopping thrombocyte aggregation.

Plasma exchange should be initiated as early as possible, ideally within the first 4–8 h after clinical diagnosis (78, 110).

For conducting PEX, usually 1–1.5 plasma volume exchanges are done every 24 h, which equates to around 40–60 ml per kg body weight. In an average adult, approximately 3–4 liters of plasma are exchanged per session. Patient plasma is replaced with donor FFP or solvent detergent treated plasma (SDP).

The current body of evidence has not shown differences for the use of FFP or SDP (111–114).

From a practical standpoint, FFP is most commonly used and readily available.

Plasma exchange is performed daily until the patient achieves clinical response, defined by normalization of platelets (>150 × 10^9^/L) (78, 115), LDH below 1.5 × ULN and no persistent or new symptoms of organ ischemia (75).

ADAMTS13 levels are not included for the definition of clinical response and extended PEX after clinical response is no longer recommended practice (75). Tapering PEX (reduced frequency or volume) has not led to lower relapse rates (116, 117).

The median number of days of PEX is 7–10 in a typical iTTP episode. If platelet count does not respond after approximately 4–7 days the TTP is considered refractory.

In patients with cTTP, sole infusion of FFPs is sufficient to replace ADAMTS13. As no autoantibodies are present, there is no indication for plasma removal. However, treatment recommendations have now changed with the availability of recombinant ADAMTS13 (86).

Along with PEX, high-dose corticosteroids are applied in iTTP with caplacizumab recommended in conjunction with PEX to block UL-vWF from platelet aggregation (118, 119).

Although PEX is considered a safe procedure, complications can arise from apheresis catheter placement (bleeding, pneumothorax), hemodynamic fluctuations, allergic reactions to plasma, hypocalcemia from citrate anticoagulation and possibly transfusion-related acute lung injury (TRALI).

Volume status must be monitored, since plasma is a colloid so it can cause fluid overload.

Plasma exchange can also change coagulation factor concentration including fibrinogen, sometimes leading to prolonged aPTT/PT after multiple sessions, but bleeding risk is mitigated through effective increment in thrombocytes during the treatment course.

Overall, PEX is very effective and improves platelet counts in a short interval of around 2–3 days of therapy in non-refractory patients. Critically, mortality has improved from >90% to <10% with the use of PEX. However, access to urgent PEX can be a limiting factor and prognosis worsens when PEX is delayed. In such cases, e.g., if PEX cannot be performed in the hospital of first admission, some guidelines consider infusion of plasma (78) to replenish ADAMTS13 before or during the patient’s transfer to a specialized center.

### Immunosuppressive therapy

Immunosuppressive therapy is an elementary pillar in the treatment of iTTP (summarized in Figure 1). As for all conditions of immunosuppression, and especially in the context of rituximab-based treatment, chronic infections and specifically infection with hepatitis B, C and HIV need to be excluded (serology and/or PCR-based assays) before initiating treatment. Prophylactic or concomitant antiviral therapy may be used as indicated.

High-dose steroids are a cornerstone in all cases of iTTP to suppress the production of anti-ADAMTS13 antibodies. Most frequently, a regimen of 1 g intravenous methylprednisolone daily over 3–5 days or prednisone at ∼1 mg per kg body weight are utilized (78, 105).

However, higher steroid doses (10 mg/kg/day over 3 days followed by 2.5 mg/kg/day vs. a standard dose of 1 mg/kg/day) were associated with a significantly higher frequency of remission (120).

Steroids help to reduce inhibitor titers in the circulation in conjunction with other immunosuppressive measures and PEX. An often-applied sequence in daily practice is an intravenous treatment start with methylprednisolone to assure high doses and systemic exposure and a subsequent switch to oral maintenance when parameters improve (78, 105).

Steroid treatment is maintained throughout PEX and then slowly reduced over 3–4 weeks once remission is stably achieved. Tapering steroids helps to avoid a rebound, and provides an interval until rituximab-mediated effects fully manifest. Putative steroid side effects need to be considered (hyperglycemia, infection risk, psychosis, etc.) and managed accordingly (78, 105).

Of note, the use of steroids along with PEX has constituted standard clinical practice for decades. However, contemporary randomized clinical trials may help to better inform on optimal dosing schedules and sequence, especially in the context of new treatments (78, 105).

Rituximab is another elementary pillar in the treatment regimen for iTTP. This monoclonal antibody against CD20 on B cells is used to deplete B cells as the source of autoantibody production. Although in some areas it may not be formally approved by authorities and could constitute an off-label use (as is the case in Germany, while insurance still covers this indication) rituximab is recommended by the majority of treatment guidelines and is used in iTTP cases during the acute phase or shortly after.

Rituximab added to PEX and steroids was shown to mitigate relapses, improve response and rates of remission and reduce mortality (121–124).

Initiation of rituximab is recommended within 72 h of diagnosis with 375 mg/m^2^ delivered intravenously (commonly for four doses), either every 3–4 days or once weekly for 4 weeks (78, 125).

Importantly, rituximab is partially removed by PEX (126) and should be given directly after the session (127).

Its efficacy in removing the B cell source of anti-ADAMTS13 autoantibodies has been confirmed in the specific context of iTTP (126).

Although the established mode of application for rituximab is by intravenous infusion, a recent study reported results of subcutaneous preemptive rituximab treatment in patients in clinical remission of iTTP displaying severe ADAMTS13 deficiency during follow-up (128). Obinutuzumab and ofatumumab have been used in cases of insufficient rituximab response, refractory disease or an inability to receive rituximab due to severe allergy (129–131).

Other immunosuppressive agents have also been employed, such as cyclophosphamide (132), an alkylating agent commonly used in severe lupus or other autoimmune conditions.

Vincristine, a vinca alkaloid, which can reduce antibody producing cells, has been used in refractory iTTP (133–135).

Due to its toxicity, and lack of specificity, better options should be prioritized from the current treatment landscape (78).

Cyclosporine has also been used in cases of refractory iTTP (136, 137) as has mycophenolate mofetil (138, 139). However, broad immunosuppression, particularly with prolonged duration, is associated with an increased risk of malignancies, severe infections, and hematologic toxicities.

In contrast, more targeted treatment strategies involving daratumumab, a CD38-targeting antibody, and bortezomib, a proteasome inhibitor, which are highly effective in targeting malignant plasma cells in myeloma, may be more useful and are being assessed for iTTP. For example, there is an increasing number of reports of effective use of bortezomib in patients with rituximab refractory iTTP. Mechanistically, bortezomib most likely works by eradicating long-lived plasma cells, which produce the anti-ADAMTS13 antibodies (140–142). Doses used in the treatment of myeloma are equivalent to those reported for use in iTTP (1.3 mg/m^2^ subcutaneous injection, days 1, 4, 8, 11 of a 28-day cycle) (142).

Daratumumab has also shown very promising activity in refractory cases (143, 144).

### Caplacizumab, an anti-UL-vWF nanobody treatment

Caplacizumab has been a breakthrough in iTTP therapy. It is a bivalent humanized single-domain antibody (nanobody) which targets the A1 domain of the vWF and blocks binding of platelet glycoprotein Ib-IX-V complex. By blocking the vWF A1 domain, caplacizumab prevents the formation of UL-vWF/platelet strings and consecutive microthrombi formation, independent of ADAMTS13 activity (145).

Using this approach, clotting is blocked until effects of PEX and immunosuppression can take effect.

Notably, caplacizumab does not act against the underlying autoimmune mechanism, nor is it replacing ADAMTS13 or reducing UL-vWF. These pathophysiological mechanisms are addressed through immunosuppression and PEX.

Caplacizumab was tested for safety and efficacy, with time to recovery of the platelet count above 150 × 10^9^/L as the primary endpoint, in the phase II TITAN-study (118) and the phase III HERCULES-study (119).

It was approved in 2018 (EU) and 2019 (USA) as the first targeted drug for iTTP. Notably, it is not indicated nor approved for use in cTTP.

A meta-analysis including the TITAN and HERCULES trials reported a significantly reduced risk of refractory disease, shortened time to response, duration of PEX use, and improved exacerbation rates at the expense of increased bleeding risk for the use of caplacizumab (146).

A recently published international multicenter retrospective cohort study (The Capla 1000+ project) assessed outcomes for *n* = 1,015 evaluable patients treated for iTTP with caplacizumab in conjunction with PEX and immunosuppression (corticosteroids alone or together with rituximab) compared to *n* = 510 patients treated only with PEX and immunosuppression (corticosteroids alone or together with rituximab) (1).

In this analysis the use of caplacizumab was associated with significantly higher rates of 3-month survival of 98.5% compared to caplacizumab-free treatment (94% 3-month survival after first PEX (*p* < 0.001)). Notably, the improvement in the 3-month survival rates was found to be independent of the use of rituximab. Caplacizumab was further significantly associated with lower rates of refractoriness (1% vs. 10.1%, *p* < 0.0001) and lower exacerbation rates (4% vs. 32%, *p* < 0.0001), but did not significantly change time to ADAMTS13 activity ≥ 20% from first PEX (*p* = 0.07) (1).

Today, adding caplacizumab to standard therapy with immunosuppression and PEX in acute iTTP events is recommended by major guidelines (105).

Caplacizumab, marketed under the name Cablivi, is given in a two-step dosing approach. The initial application is conducted with a 10 mg intravenous bolus before the first PEX to quickly achieve high drug levels. Subsequently, 10 mg per application are administered subcutaneously every day directly after PEX, to avoid caplacizumab removal. When PEX is stopped, caplacizumab should be given at around the same time daily. Treatment is generally continued for 30 days after the last PEX. If at 30 days after PEX the ADAMTS13 activity is still diminished, most experts extend the use of caplacizumab (in conjunction with other measures) for an additional 2–4 weeks to secure remission and protect against early relapse (78, 86, 92, 119).

Favorable outcomes have been reported in case series when ADAMTS13 activity has reached >10%. Conversely, activity <10% is associated with increased rates of disease exacerbation or relapse (147).

Stopping caplacizumab early (before day 30 after PEX) based on platelet count recovery was associated with high rates of exacerbation. Before ending treatment, ADAMTS13 activity of ≥20% (partial remission) (148, 149) for at least two consecutive weeks has been suggested (149). However, another study reported no TTP recurrences when ADAMTS13 activity was above 10% (147). A later study suggested two consecutive ADAMTS13 measurements above 10% (after discontinuation of PEX and in the absence of persisting strong TTP triggers) before stopping caplacizumab (150).

While caplacizumab is given during PEX, platelet counts usually normalize rapidly. If platelets drop again while on therapy, it implies either an inadequate treatment, which could derive from refractory disease, or a concurrent problem, such as sepsis or DIC.

As noted, caplacizumab does not treat the underlying ADAMTS13 depletion. Correspondingly, the key differentiator of response is ADAMTS13 activity during caplacizumab treatment and extending caplacizumab application while intensifying the immunosuppression needs to be considered when ADAMTS13 activity remains low.

In routine settings, ADAMTS13 is checked at least once weekly after PEX has been stopped, to guide caplacizumab duration. After confirming ADAMTS13 recovery with levels >20% activity and rising (ideally normal levels are achieved), caplacizumab can be stopped (78, 148, 149). The main side effect of caplacizumab is increased bleeding risk due to inhibition of vWF-platelet binding (78, 118, 119). Most bleeding events are mucocutaneous (epistaxis, gingival bleeding, menorrhagia), while the risk of major bleeding is reported to be comparably low (118, 119, 146).

Of note, patients showing major bleeding were reported to be older than patients with non-major bleeding or no adverse events (57 (Q1–Q3, 47–63.5) vs. 45 (Q1–Q3, 33–58) years old, *p* = 0.006) (1).

Importantly, patients with active bleeding or conditions associated with an increased bleeding risk, including the need for continued anticoagulant treatment, have been excluded from the TITAN and HERCULES studies, which needs to be taken into consideration when discussing bleeding rates (118, 119).

In summary, patients with increased bleeding risk, older age or those developing bleeding during treatment need to be carefully assessed for individual risk and bleeding signs before and during caplacizumab treatment. Non-mandatory interventional measures, which may increase bleeding risk, should be delayed if possible.

In cases of severe bleeding, the successful substitution of vWF-containing concentrates has been reported (119).

Reduced-frequency regimens, such as alternate-day dosing of caplacizumab (151), may offer a promising strategy to reduce bleeding risk and treatment costs. However, their safety and efficacy remain to be confirmed prospectively.

### Plasma exchange-free iTTP management

Novel approaches in managing iTTP have become possible through the advent of caplacizumab, which now allows the exploration of regimens without PEX (summarized in Figure 1).

Since caplacizumab immediately prevents the further formation of thrombi, caplacizumab with immunosuppression using steroids and rituximab might enable the omission of PEX in patients who are otherwise stable.

It remains to be explored if PEX-free caplacizumab treatment along with immunosuppression shows favorable safety and efficacy. Such treatments may be of especially high value for scenarios when PEX is not immediately available. Nevertheless, currently, PEX remains the standard of care and omission of PEX is an off-label approach which requires careful judgment and individual patient assessment by experts in the field.

While PEX-free treatment remains to be explored in randomized trials, it has been practiced by some experts in suitable patients with considerable success (152–154).

### Managing refractory or exacerbated iTTP

Some patients fail to improve or worsen after initial improvement despite standard therapy and are therefore considered refractory or to have exacerbated iTTP. However, the development of refractory iTTP is a very rare phenomenon.

Novel treatment options have led to a revision of the outcome definitions for iTTP (75). Clinical response is now defined as a sustained platelet count ≥ 150 × 10^9^/L, LDH <1.5 times ULN and no clinical evidence of new or progressive ischemic organ injury (75).

In contrast, clinical exacerbation is defined as a decrease in platelet count to <150 × 10^9^/L (with other causes of thrombocytopenia excluded), with or without clinical evidence of new or progressive ischemic organ injury, occurring after a clinical response but before clinical remission, within 30 days of stopping therapeutic PEX or anti-vWF therapy (75).

Refractory iTTP has not been revised from the 2017 consensus and remains defined as persistent thrombocytopenia, lack of a sustained platelet count increment or platelet counts of <50 × 10^9^/L and a persistently raised LDH level (>1.5 × ULN) despite five PEX and steroid treatment (155, 156).

If refractoriness or exacerbation is suspected, several steps are recommended to be followed.

Most importantly, reaffirmation of the correct diagnosis is mandatory and other TMAs need to be ruled out. An initial ADAMTS13 activity <10%, along with inhibitor testing is essential to reconfirm the initial diagnosis of iTTP. In addition, triggers for iTTP should be identified and, where possible, treated, as they may impair response to the chosen treatment (150).

Further, other causes leading to thrombocytopenia during PEX, such as catheter-associated infection and sepsis, drug-induced thrombocytopenia or viral infection, need to be ruled out first.

The rates of refractoriness and exacerbation have considerably decreased with the use of caplacizumab and standard immunosuppression compared to historic controls and were reported with 1% vs. 10.1% (*p* < 0.0001) and 4% vs. 32% (*p* < 0.0001) (1).

If not already used, addition of caplacizumab is the most obvious choice in cases with iTTP as it was reported to decrease rates of suboptimal response after treatment initiation (1, 157).

Further, effective immunosuppression, involving rituximab, needs to be ensured as the use of rituximab has been shown to improve response rates and shorten the time to platelet recovery in rituximab-naive patients (158).

Further, increasing corticosteroid doses is an effective option to be directly implemented (120). As another option, intensity of PEX can be increased. Some centers will conduct two PEX cycles per day in refractory TTP. Alternatively, the amount of plasma volume exchange can be increased during a single PEX cycle per day.

If these measures do not lead to an improvement in the clinical situation, increasing immunosuppression with previously discussed therapeutics (e.g., bortezomib, daratumumab, cyclophosphamide) can be implemented after confirming the presence of an inhibitor.

## Management of congenital TTP (cTTP)

The cornerstone of treating hereditary TTP is replacement of the missing ADAMTS13 and managing or preventing triggers provoking episodes of TTP. Until recently, the mainstay for cTTP patients in remission was regular plasma infusions or a watch and wait strategy (105).

Fresh frozen plasma infusions supply ADAMTS13 through which the activity level can be increased above the critical 10% threshold to prevent TMA episodes. Typically, 10–15 ml plasma per kg body weight is infused every 1–3 weeks (105), but adjustment may be needed based on clinical and lab monitoring.

Some patients require more frequent infusions, possibly weekly, if they present with more severe disease. Clinical symptoms like headache or fatigue can indicate a new episode and might prompt investigation and extra infusions (159).

Recently, recombinant ADAMTS13 (rADAMTS13) was shown to dramatically improve the treatment options for cTTP patients, both regarding efficacy and convenience (160).

Recombinant ADAMTS13 is produced in cell culture and marketed as apadamtase alfa (brand name Adzynma). It was first approved in 2023 in the US and in 2024 in Japan and the EU and constitutes the first specific therapy for cTTP, indicated both for prophylaxis and on-demand treatment for acute episodes in cTTP patients (86, 161).

It was shown that rADAMTS13 prevents TTP episodes effectively and is well-tolerated. The standard schedule for prophylactic dosing is 40 IU/kg body weight once every other week but may be adjusted to weekly dosing if needed (161). Neutralizing antibodies against rADAMTS13 should be considered if routine monitoring shows that ADAMTS13 activity remains below expected levels or if acute exacerbations occur despite appropriate dosing.

Current guidelines now strongly recommend rADAMTS13 over plasma infusions as a maintenance therapy in cTTP patients in remission (86).

In previously undiagnosed patients, the immediate treatment is applied as for iTTP until the absence of an ADAMTS13 inhibitor and the results of genetic testing for an *ADAMTS13* mutation confirm the correct diagnosis.

## Special situations

### TTP in pregnancy

Thrombotic thrombocytopenic purpura poses unique challenges in pregnancy, both for the acquired and the congenital form.

About 10% of TTP presentations are pregnancy-associated, with an incidence of about 1/200000 pregnancies (28, 68, 71).

Other TMAs need to be rapidly considered and eliminated in the differential diagnosis in this specific context, which warrants interdisciplinary consultation due to the very challenging situation. Also under physiological conditions, blood counts show relevant changes and therefore a decrease in platelets or hemoglobin may not derive from TMA/TTP but needs stringent further evaluation and control (97).

Recent data from the French Registry for TMA identified pregnancy-onset TTP to be predominantly immune-mediated (iTTP 48.1%, uTTP 25%, and cTTP 26.9%) (162).

These findings also confirm the previously reported high frequency of cTTP in the context of pregnancy (28, 63, 67).

The majority of TTP cases occur in the third trimester or postpartum (65%) (162).

Women with a history of iTTP or diagnosed cTTP are at increased risk for relapse or other pregnancy-associated complications (26, 163).

Monitoring and prophylactic measures therefore need to be ensured according to current guidelines (86) and the patient should be monitored by a multidisciplinary team consisting of hematologists and obstetricians experienced with high-risk pregnancy. A referral to a specialized center prior to an acute TTP episode, ideally in the preconception period, is best practice.

For treatment of iTTP during pregnancy, corticosteroids and PEX are used, with treatment principles remaining unchanged. PEX appears safe, however it needs careful monitoring (e.g., for volume, coagulation, blood pressure, etc.) (86, 97).

Rituximab is not routinely given in pregnancy unless absolutely necessary. Rituximab can cross the placenta and lead to B cell depletion in the fetus for months, delaying the neonatal period of immune maturation (164).

Caplacizumab is also not recommended for use during pregnancy as reliable data on risks or benefits are insufficient. However, use has been reported in the context of exacerbation and refractory iTTP (165, 166) and can be considered while its future role remains to be evaluated in this context (167). Importantly, an increased likelihood of hemorrhage has to be considered.

In cTTP, plasma infusions are an established prophylactic and therapeutic option, while rADAMTS13 has been used successfully during pregnancy, even in some cases of iTTP (168–170).

Patients with a preceding episode of iTTP or diagnosed cTTP should be monitored for ADAMTS13 activity, ideally before conception, during pregnancy, after delivery and while receiving prophylactic treatment. Even in the absence of TTP associated clinical or laboratory alterations, monitoring is essential.

Timing of delivery is dependent on gestational age and maternal stability; however, if TTP is uncontrolled or if the situation is urgent and distinction from preeclampsia/HELLP syndrome remains unclear, delivery may be required promptly and PEX can be initiated and continued in parallel, including over the postpartum period (171).

Outcome and prognosis of TTP in pregnancy have improved over recent years and many women can have a successful pregnancy. However, pregnancy in TTP patients has a considerable risk for both the mother and the fetus, with increased miscarriage, complications and mortality (9, 67, 86). Outcomes depend on the type of TTP, possibilities to monitor the patient, means of prophylactic treatment and opportunity to secure specialized consultation.

While TTP is not teratogenic, uteroplacental insufficiency is a problem, which is why close fetal monitoring needs to be secured.

Due to the high risk of relapse during pregnancy, contraception is important in women with TTP who are not planning pregnancy, as prophylactic measures and monitoring should be in place before conception.

### Childhood TTP

Childhood cTTP may present at birth or in early infancy. Jaundice, high LDH and thrombocytopenia in the neonate should raise suspicion of congenital TTP, as prompt ADAMTS13 replacement can be life-saving. As the child grows, chronic plasma infusions can be challenging due to volume but rADAMTS13 was recently approved for use in pediatric patients (160).

If a patient’s diagnosis is confirmed by *ADAMTS13* sequencing, genetic testing can be offered to other family members. Comprehensive consultation should be provided to affected individuals, parents and other family members, including those who have not yet received genetic testing, to raise awareness for TTP triggers and clinical signs of a TTP episode (9, 78, 86, 172, 173).

Patients with cTTP need lifelong prophylaxis and monitoring. They should carry a medical alert card and be educated on further steps if symptoms, suggestive of a TTP episode, occur. Potential triggers, such as infections, should be mitigated through routine vaccination and high-risk situations, such as surgery, carefully managed by experienced multidisciplinary specialists (9, 78, 86, 161, 172, 173).

In this context, the scientific community is increasingly focused on how emerging therapies, such as recombinant ADAMTS13, together with improved disease monitoring, may further enhance life expectancy and quality of life, and potentially reduce the burden of lifelong prophylactic treatment (9, 173).

In contrast to cTTP, iTTP is rare in pediatric populations and particularly uncommon in younger children. While caplacizumab has become a cornerstone of iTTP treatment in adults, it is not approved for children <12 years or less than 40 kg in most jurisdictions. Evidence for its use in younger children is scarce (174) and optimal dosing strategies in this population have not yet been clearly established (175).

Treatment decisions must therefore be individualized, taking into account available evidence, disease severity and expert clinical judgment. Given the complexity and rarity of pediatric iTTP, these patients should be treated in specialized centers for guiding treatment decisions, monitoring bleeding risk and acquiring formal institutional or ethics approval for off-label therapies, including caplacizumab.

It is important to note that the pediatric population also presents specific challenges regarding the use of diagnostic tools established for TTP in adults. The PLASMIC score, which was developed and validated in adult cohorts, cannot be directly extrapolated to children. Several components of the score, especially serum creatinine, are age-dependent and may lead to misclassification. Adapted tools, such as the “PLASMICkid” score (176) are currently under investigation but remain insufficiently validated.

Consequently, PLASMIC-based risk stratification in children should be interpreted with caution. Prompt ADAMTS13 activity testing and expert clinical assessment remain the cornerstone of diagnosis. Treatment decisions should not be delayed based on adult-derived scoring systems.

### TTP in the elderly

Manifestation of TTP is possible at any age, including in elderly patients, although the incidence is lower than in younger adults.

Older iTTP patients are reported to more frequently present with atypical neurological symptoms (delirium with behavioral disturbance) which is why the diagnosis is delayed in comparison with younger patients (177).

During the differential diagnostic work-up, alternative causes of TMA, including cancer-associated MAHA/TMA, need to be considered, as the incidence of malignancy increases with older age. The most frequent associations have been reported for metastatic adenocarcinomas of the lung, breast, prostate and stomach (178, 179).

Cancer-related MAHA/TMA is considered a paraneoplastic manifestation and is primarily managed with tumor-directed therapy. In most cases, ADAMTS13 activity is not severely reduced and the clinical presentation differs from classical immune-mediated TTP. However, rare cases with severe ADAMTS13 deficiency and a more typical TTP-like presentation have been reported (178, 179).

Thrombotic thrombocytopenic purpura treatment and surveillance will follow the same therapeutic principles as outlined earlier, but patients of higher age usually have more underlying comorbidities, for example of the cardiovascular or renal system, that may put these patients at particularly high risk of complications such as myocardial infarction, stroke or renal insufficiency (1, 177).

Patients with underlying comorbidities and acute complications may tolerate PEX, and corresponding risk of fluid overload, less well. In this context, closer monitoring needs to be facilitated and the PEX schedule may be modified (less volume exchange, altered frequency, blood pressure control, etc.).

Among older patients the 1-month and 1-year mortality are reported to be significantly higher and increased for every 10-year range of age (177). Long-term survival rates were reported to be lower in comparison to non-iTTP geriatric individuals (177).

Success of rehabilitation may be decreased and recovery delayed, requiring more intensive or longer support. Long-term neurologic and cardiac deterioration with persisting symptoms may impact the patient’s autonomy in daily life and older iTTP survivors were reported to be less likely to stay at home after discharge from hospital (177).

While the use of caplacizumab has improved outcomes in elderly patients, age was the factor most associated with 3-month mortality and major bleeding was more common in elderly patients (1).

In addition, the use of immunosuppressive treatments requires careful consideration and follow-up, considering the increased risk of malignancy and the potential to exacerbate age-related conditions such as immunosenescence, sarcopenia and decreased bone-density, which may warrant prophylactic measures.

### HIV-associated TTP

Uncontrolled HIV infection has historically been a high risk for developing TTP (180). HIV-TTP is associated with high morbidity and mortality, especially in poorer countries with reduced access to optimal treatment (180).

Pathomechanistically, endothelial dysfunction induced through inflammation and complement activation is regarded as a prominent factor for developing TTP and the generation of UL-vWF (181–183).

ADAMTS13 levels in HIV-associated TTP were found to be reduced and autoantibodies were detectable (39, 184, 185).

However, the exact pathomechanism of TTP in HIV patients is not yet established and diagnostic criteria are not as clearly defined due to variable levels of ADAMTS13 and autoantibodies (39, 183, 186).

Daily PEX remains the cornerstone of treatment, as previously described, also in HIV patients (78, 184, 187). The addition of an immunosuppressive therapy has to be considered but requires additional diagnostic steps to rule out active infections. Prophylactic antimicrobial therapy should be initiated as recommended in HIV patients and with regard to the immunosuppressant used (188, 189).

The use of caplacizumab and rituximab was reported as effective and led to a rapid and complete remission in the reported cases (190).

In patients with established antiretroviral therapy, adherence should be secured and treatment optimized while in treatment-naive patients, adequate antiretroviral therapy needs to be initiated.

Maintaining treatment is a central aspect to prevent relapse (184, 191).

Preexisting HIV-associated alterations with regard to the hematopoietic capacity also need to be considered when evaluating treatment response in these patients (192).

## Outcomes and prognosis

### Survival

With adequate and timely treatment, the vast majority of patients with acute TTP survive.

Mortality during an acute iTTP episode is reported to be at 6.6% within 30 days (193).

Another study reported in-hospital mortality in patients with TTP receiving PEX of 11.1%. Predictors of in-hospital mortality included older age, myocardial infarction and heart failure and acute cerebrovascular disease (194).

Other studies reported similar predictors of poor outcome. These involved higher age, serum LDH levels more than 10 × ULN and severe cerebral involvement at presentation (195). Also, troponin levels above the ULN and Glasgow Coma Scale (GCS) of 14 or less [with a reported 6- (troponin) and 9-fold (GCS) increase in mortality] were identified (196).

Low ADAMTS13 antigen levels and high anti-ADAMTS13 antibodies were correlated with higher mortality in one study, associated with increased cardiac and neurological involvement (196).

In a study of patients enrolled in two US TTP registries and followed for a median of 4.5 years, patients experiencing an iTTP episode showed a 1.8 times higher mortality rate in comparison to people of the same race, age and sex without an iTTP episode (197).

The reported three leading causes of death were cardiovascular disease (27.6%) and relapsed iTTP (27.6%), followed by malignancy (20.7%), besides other reasons (197).

Another study identified an increased risk of ischemic stroke in patients with iTTP in remission, which was associated with low ADAMTS13 activity (198).

Notably, novel treatment with caplacizumab and rADAMTS13 may considerably change these mortality rates. As reported in one large retrospective study, the 3-month mortality rate was 4.2-fold lower for patients receiving caplacizumab than controls (1).

### Relapse, prevention, and long-term follow-up

Approximately 40% of iTTP patients relapse after 5 years of initial treatment, when both clinical and ADAMTS13 relapse are considered (4). Previous studies reported similar numbers, while the clinical relapse rate has declined over time with more frequent monitoring and preemptive use of rituximab (77, 199). In particular, ADAMTS13 relapses (activity of <20% without thrombocytopenia) were reported to respond to anti-CD20 therapy (96% responders) achieving ADAMTS13 activity of >20% (4).

Many relapses occur within the initial years after the first episode, but late relapses after more than 10 years can happen, reminding of the lifelong need for vigilance.

Relapses now tend to have a better outcome as monitoring is closer and awareness for signs and symptoms is high, which helps to start treatment at the earliest timepoint possible.

Some factors can flag an increased risk of relapse. These comprise a decreased ADAMTS13 activity after recovery, the presence of an inhibitor after reaching remission, HLA characteristics and ethnic background (e.g., a higher incidence and relapse rate has been reported in patients with African origin) (4, 7, 30, 77).

Education on symptoms and endorsement for early presentation at the specialized center, involving the patient, family members and general practitioner, are important measures to facilitate awareness and early action. For patients with iTTP in clinical remission, routine clinical and laboratory surveillance is recommended, initially on a monthly basis, followed by quarterly assessments and eventually once or twice per year, depending on the individual disease course. Laboratory evaluation includes ADAMTS13 activity and inhibitor levels, complete blood count, LDH, basic chemistry and hemolysis parameters (86, 200).

In a historical cohort of 23 patients with persistently undetectable ADAMTS13 activity (<10%) who did not receive preemptive therapy, a clinical relapse rate of 74% was observed after a median follow-up of 7 years (23).

In contrast, observational registry data from 17 patients without ADAMTS13-guided intervention, who had at least one remission ADAMTS13 measurement with activity <10%, showed a median time to relapse of 5.4 years (24).

However, these findings should be interpreted in context, as differences in cohort characteristics, surveillance intensity and management approaches, particularly the use of preemptive rituximab, limit direct comparability.

Preemptive use of rituximab in patients with severely reduced ADAMTS13 activity can help to considerably reduce relapse rates as previously reported (23).

Using rituximab for patients in clinical remission but low ADAMTS13 activity has been suggested as a strategy to prevent clinical relapse and remains a valid approach (86, 105).

As previously detailed in this review, conformation of ADAMTS13 has gained interest in this context as an open ADAMTS13 conformation can indicate subclinical iTTP and may be used during follow-up to monitor the chance of relapse (50, 51).

More recently, changes in the vWF multimer ratio have gained attention as a possible marker of disease activity in iTTP (201).

After surviving an acute episode of TTP, follow-up should initially be weekly or twice a week until platelet count and LDH are stable without therapy. After that, monthly visits for 3–6 months including physical examination, neurologic testing and laboratory assessment including ADAMTS13 activity should take place.

Intervals can then be extended to 3 months for 1–2 years. Long-term follow-up should happen every 6–12 months (all visits should include ADAMTS13 testing). Patients need to be educated and present on their own initiative if symptoms occur.

Conversation with the primary care physician needs to cover critical laboratory values (hemolysis parameters and complete blood counts) and symptoms which need to receive special attention and trigger alert and quick consultation with the specialist. Primary care physicians also need to be made aware of potential triggers such as new drugs, autoimmune diseases, pregnancy and others.

Management of long-term complications is an important pillar in the treatment of TTP patients after recovery from an acute episode. All patients need to have neurocognitive evaluation if CNS involvement was present, along with screening for depression and posttraumatic stress disorder. In these cases, either inpatient or outpatient neurologic rehabilitation or psychological support should be provided. Support groups can provide relief for traumatized patients.

Due to the risk of cardiovascular complications, hypertension and stroke, risk factors should be aggressively managed, especially stringent blood pressure control, cessation of smoking, dietary restrictions, regular activity and weight loss (202).

## Conclusion and future directions

Recent developments in TTP treatment both with regard to iTTP and cTTP are a clinical success story with repeated breakthrough treatments and improvement of outcomes. The pace of progress in diagnostics, treatment and surveillance of affected patients has accelerated with rapid advances in recent years. Especially clinical applications of caplacizumab and rADAMTS13 represent the natural evolution of an improved understanding of disease mechanisms and its translation into therapeutic strategies. Further improvements and development of TTP treatments can be expected in the near future and multiple opportunities exist:

Caplacizumab has a short half-life, necessitating daily injections. Its mechanism of action, blocking the interaction between von Willebrand factor and platelets, results in a transient functional impairment of primary hemostasis, which may increase the risk of mucocutaneous bleeding. Individualized dosing strategies may help to mitigate this risk (151).

Next-generation vWF-targeting therapies are therefore being explored, aiming to optimize pharmacokinetics while maintaining efficacy and reducing bleeding risk.

Future directions include next-generation immunosuppressive and B cell-targeting strategies, such as obinutuzumab or proteasome inhibitors (129, 130, 140, 142–144), and may also involve novel modalities for autoantibody clearance, including FcRn blockade, which has shown promise in other IgG-mediated autoimmune disorders such as ITP (203).

In addition, the use of rADAMTS13 may be further personalized in cTTP, for example by considering residual enzyme activity.

Further, there is also a need to evaluate its use in the context of pregnancy in randomized studies.

While plasma-free treatment strategies are gaining interest and are already applied in specialized centers, clinical trial data to support their broader implementation are still emerging (92). Standardized criteria for safely omitting plasma exchange remain to be defined.
